# Innovations in Molecular Biomarkers and Biomaterial-Based Immunotherapies for Head & Neck Cancer

**DOI:** 10.1007/s40137-024-00386-z

**Published:** 2024-02-28

**Authors:** Sarah Anne Wong, Victoria A. Manon, Simon Young, Chi T. Viet

**Affiliations:** 1 School of Medicine, Orthopaedic Trauma Institute, University of California San Francisco, 2550 23rd St., Bldg. 9, 3rd Floor, San Francisco, CA 94110 USA; 2 Bernard and Gloria Pepper Katz Department of Oral and Maxillofacial Surgery, The University of Texas Health Science Center at Houston School of Dentistry, 7500 Cambridge Street, Suite 6510, Houston, TX 77054 USA; 3 Department of Oral and Maxillofacial Surgery, Loma Linda University School of Dentistry, 11092 Anderson St., Loma Linda, CA 92350 USA

**Keywords:** Biomarker, Immunotherapy, Oral cancer, Head and neck cancer, Biomaterials, Non-invasive biopsy

## Abstract

**Purpose of Review:**

Oral squamous cell carcinoma (OSCC) survival rates have remained stagnant due to a lack of targeted therapies and diagnostic tools. Patient risk is currently determined solely through clinicopathologic features, primarily tumor staging, which lacks the necessary precision to stratify patients by risk and accurately dictate adjuvant treatment. Similarly, conventional OSCC therapies have well-established toxicities and limited efficacy.

**Recent Findings:**

Recent studies show that patient risk can now be assessed using non-invasive techniques, at earlier time points, and with greater accuracy using molecular biomarker panels. Additionally, novel immunotherapies not only utilize the host’s immune response to combat disease but also have the potential to form immunological memory to prevent future recurrence. Localized controlled-release formulas have further served to reduce toxicity and allow the de-escalation of other treatment modalities.

**Summary:**

We review the latest advances in head and neck cancer diagnosis and treatment, including novel molecular biomarkers and immunotherapies.

## Introduction

Head and neck squamous cell carcinoma (HNSCC) is the sixth most common cancer globally, with more than 60,000 annual new cases in the U.S. alone [1]. HNSCC can be divided into distinct sub-types, each featuring unique anatomic locations, etiology, patient demographics, and molecular profiles. Of these, oral squamous cell carcinoma (OSCC) remains one of the most deadly. There have been few improvements in patient outcomes in recent years, and OSCC incidence is currently on the rise. In fact, in the last 20 years, OSCC incidence has increased by two-thirds, predominantly in young patients, resulting in 400,000 new annual cases worldwide and 30,000 new cases in the U.S. each year [2]. Half of these individuals will die of this disease, resulting in approximately one death per hour [3•]. This high mortality is combined with significant morbidity due to conventional OSCC treatment, which often results in functional and cosmetic deformities that reduce patients’ ability to eat, speak, taste, and relate to others. This significant burden necessitates the development of novel therapies and diagnostic tools.

The era of personalized medicine began in 2001 with the completion of the human genome project. The goal was to use the genetic code to develop unique biomarker panels that could identify a patient’s individual cancer risk as well as the aberrant molecular pathways causing disease. These dysregulated pathways could then be addressed with targeted therapies. This method has proved successful in several cancer fields, particularly breast cancer where commercially available genomic tests are currently used to guide treatment, predict patient risk of recurrence, and improve patient survival, especially in young women with metastatic disease [4].

Biomarkers have also proved useful in diagnosing specific HNSCC sub-types, particularly oropharyngeal cancer (OPSCC). Since the majority of OPSCC cases (> 70%) are caused by the human papilloma virus (HPV), it has become standard-of-care to test patients for over-expression of *p16*, a biomarker of HPV infection, and to risk-stratify them accordingly [5]. HPV-positive OPSCC patients have significantly higher survival (82.4% 3-year survival rate) compared to their HPV-negative counterparts (57.1%) [5]. Recently, HPV-positive OPSCC patient survival has increased as high as 90%, even with treatment de-escalation [6•]. In contrast, HPV does not significantly contribute to OSCC etiology or prognosis, even in young non-smokers, and no OSCC biomarker equivalent to *p16* exists [7].

The field of OSCC biomarker research has seen significantly fewer advancements compared to that of other cancers. Molecular biomarkers that can effectively distinguish high- versus low-risk OSCC patients of the same stage have only recently been discovered. This ability to risk-stratify patients at early time points is critical since the majority of OSCC cases (80%) are diagnosed at early stages (I/II) without regional lymph node involvement or distant metastasis [8••]. Yet, despite early diagnosis, patients’ 5-year mortality risk remains at 40–60% [3•, 8••]. This high mortality is explained, at least in part, by a lack of diagnostic tools that can effectively identify patient risk and dictate appropriate treatment. Current treatment for early-stage OSCC patients is highly variable, ranging from surgery alone to surgery plus any combination of the following: elective neck dissection (END), radiation (RT), and chemoradiation (chemoRT). The selection of adjuvant therapies has been previously determined solely using clinicopathologic features such as tumor stage and grade, depth of tumor invasion, margin status, lymphovascular invasion (LVI), and perineural invasion (PNI). However, this method fails to accurately risk stratify patients and has led to unpredictable outcomes in patients. The combined use of molecular biomarker panels and clinicopathologic features has enabled more accurate assessment of patient risk and has consequently led to more appropriate selection of treatments.

Immunotherapy-based cancer treatments are becoming standard-of-care in many cancer fields, including melanoma, lung, and HNSCC [9]. In the case of HNSCC, immunotherapy is currently FDA-approved for treating recurrent and metastatic disease. Its unique mechanism of action enhances the host’s immune response, enabling the host to detect and destroy cancer cells while also developing immunological memory to prevent recurrence. Despite the theoretical superiority of immunotherapy treatments, their clinical success has remained low (15–20%). They are also known to have immune-related adverse events (irAEs), which result from often-required high dose and frequency of treatment. The toxicities experienced by patients receiving immunotherapy are likely to increase as these drugs are more frequently used and combined with other therapies. Innovative strategies that reduce immunotherapy toxicities are desperately needed [9].

The efficacy of immunotherapy drugs is dependent on the local tumor immune microenvironment (TIME). Some TIME’s, such as that for HNSCC, are known to be highly immunosuppressive. In the case of HNSCC, a large milieu of immune suppressive cells is often present, including regulatory T cells (Treg) that suppress effector T cells, myeloid-derived suppressor cells (MDSCs) that inhibit T cell activation and proliferation, and anti-inflammatory (M2) macrophages. The tumor cells themselves are also known to highly express anti-inflammatory cytokines including TGF-*β*﻿, IL-1, and VEGF as well as checkpoint molecules PD-1/PD-L1, CTLA-4, and TIM-3. Combined, this immunosuppressive TIME renders immunotherapy and conventional radiotherapy ineffective by neutralizing or killing tumor-infiltrating effector T cells. Recent developments in biomaterial-based technologies have enabled the localized delivery of immunomodulatory drugs. These drug delivery modalities include lipid nanocarriers, synthetic nano- and microparticles, implantable or injectable scaffolds, and hydrogels [10]. Preliminary data have shown that localized, controlled-release of immunotherapy drugs via biomaterials can effectively reverse the immunosuppressive TIME. The significant versatility and spatiotemporal precision of these technologies have made them highly clinically translatable. Moreover, their use holds tremendous potential in reducing toxicities from systemic exposure [10].

**Fig. 1:**
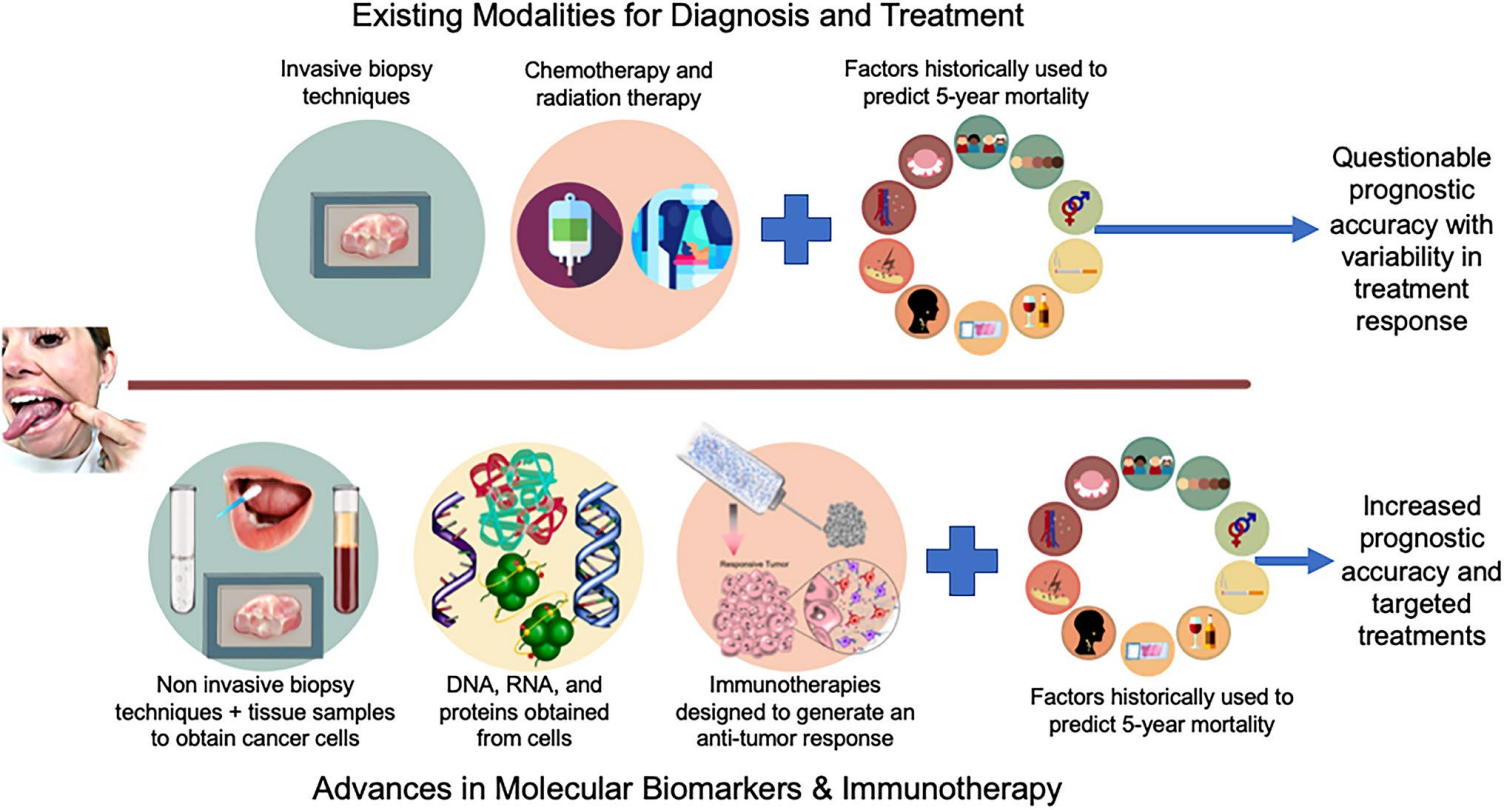
Recent advances have been made in all stages of HNSCC patient care. Whereas diagnosis and adjuvant treatment selection were previously based solely on clinicopathologic features and histologic evaluation of paraffin-embedded tissues harvested at the time of tumor resection, we are now taking steps toward accurately determining individual patient risk by combining molecular biomarker analysis with clinicopathologic features and obtaining this information prior to surgery through non-invasive biopsy techniques. Cancer treatments have seen similar advancements. Patient treatment options are no longer limited primarily to chemo- or radiation therapy, which carry notable morbidity. Rather, advances in immunotherapy now provide the potential for biological memory against recurrence, and the localized delivery of these drugs via biomaterials has enabled targeted tailoring of the immune response while reducing systemic toxicity. Together, these developments have significant potential to improve HNSCC patient outcomes

Recent advances have been made in all phases of HNSCC patient care, from innovations in patient diagnosis with molecular biomarkers to developments in cancer therapies with vaccines, immunotherapy drugs, and injectable biomaterials (Fig. 1). Here we review these latest developments and more.

## Innovations in OSCC Diagnosis Through Biomarker Research

The aim of OSCC biomarker research has been to generate a multi-gene risk score that can be used by clinicians to accurately tailor patient treatment. This has been attempted using a variety of approaches, including evaluating differences in gene expression, amplification and deletion, methylation, and microRNA (miRNA). Most studies have aimed to prevent over-treatment by predicting patients’ risk for neck metastasis and their need for END. Such metrics are desperately needed since > 20% of early stage OSCC patients do not have discernable neck metastases. With the significant mortality risk associated with occult metastases left untreated, it has become routine for surgeons to perform prophylactic neck lymphadenectomy on all OSCC patients, resulting in over-treatment in 80% of patients and significant morbidity from shoulder dysfunction, nerve damage, and lymphedema [11].

Biomarker panels initially showed limited clinical success. This was primarily because they sought to identify global markers of HNSCC. It is now clear that generating stage- and subtype-specific biomarker panels allows for more accurate molecular signatures. One example of this is a large biomarker study published in 2004, which identified a 102-gene signature by comparing changes in gene expression among patients with and without neck metastasis [12, 13]. This 102-gene signature was 86% accurate in predicting neck metastasis. However, the subsequent multi-center validation study had a 72% negative predictive value (NPV) for all stages of OSCC and OPSCC [11]. Importantly, when the patient cohort was limited to early stage (I/II) OSCC patients, the NPV increased to 89%. This significant increase not only demonstrates the importance of evaluating HNSCC by stage and sub-type but also highlights the diagnostic potential of using molecular biomarkers for risk-assessment in early-stage patients.

Early-stage OSCC patients have the greatest need and potential benefit from biomarker-based evaluation. Whereas late-stage (III/IV) OSCC patients routinely receive triple-modality therapy (surgery, radiation, and chemotherapy), treatment for early-stage patients is highly variable due to a lack of accurate and individualized metrics for risk assessment. Recent studies have sought to address this issue by identifying biomarkers that distinguish high- versus low-risk early-stage OSCC patients. Yoon et al. used miRNA patterns to predict 5-year survival in early-stage OSCC patients [3•]. When their 3-miRNA signature was combined with TNM classification and histologic grade, their risk score had a concordance (c)-index of 0.832.

Evidence now demonstrates that epigenetics (i.e., DNA methylation) plays the most prominent role in regulating OSCC progression. Numerous studies have shown that methylation leads to genomic instability and the dysregulation of genes involved in OSCC etiology [8••, 14, 15]. An epigenome-wide association study (EWAS) by Guerrero-Preston et al. demonstrated how methylation inactivates several key tumor suppressor genes in HNSCC patients [16]. However, despite the critical role of epigenetics in OSCC etiology, these epigenetic-based studies demonstrated poor prognostic success. As was the case in previous studies, this was most likely due to the use of heterogeneous study populations that included both early- and late-stage OSCC patients as well as patients of varying HNSCC sub-types. Moreover, these studies did not include clinicopathologic features and relied solely on molecular data to determine risk.

A study focused on early-stage (I/II) OSCC patients showed significant prognostic potential when using epigenetic biomarkers to predict 5-year mortality [8••]. This study used both molecular and non-molecular features to calculate patients’ mortality risk. The molecular panel consisted of methylation patterns from 12 genes, all of which had been previously linked to patient survival in other cancers. Importantly, 11 of these 12 genes had previously never been linked to OSCC. The study’s non-molecular panel consisted of the following clinicopathologic features: age, race, sex, tobacco use, alcohol use, histologic stage, grade, lymphovascular invasion (LVI), perineural invasion (PNI), and margin status. Use of patient clinicopathologic features alone to assess 5-year mortality resulted in a c-index of 0.67, which is similar to previous studies. However, this c-index increased to 0.915 when the clinicopathologic features were combined with the study’s 12-gene molecular panel [8••]. Work is currently in progress to validate this risk score using a multi-institutional cohort. Together, this data highlights the importance of using molecular biomarkers to assess patient risk and underlines its potential to reduce over-treatment in low-risk patients while ensuring adequate treatment escalation in high-risk patients.

## OSCC Diagnosis Through Non-invasive Biopsy

OSCC lesions are easily accessible for non-invasive biopsy, allowing for earlier diagnosis as well as frequent sampling to monitor for treatment response and recurrence. Whereas formalin-fixed, paraffin-embedded (FFPE) tissue samples harvested during tumor resection often result in delayed adjuvant treatment, samples harvested via non-invasive biopsy enable risk score assessment prior to surgery at the time of initial diagnosis.

Studies have used saliva, brush swabs, and circulating tumor cells (CTC) to non-invasively collect OSCC cells for diagnosis. Saliva has not proven successful since the methylation patterns between saliva and cancer tissues are highly variable [17]. However, cell samples harvested via non-invasive brush swabs have shown high concordance with cancer tissue (*r* = 0.913) [18••]. Importantly, studies have shown no significant differences in DNA yield between tissue and brush swab samples. Moreover, methylation data resulting from brush swabs can be successfully used to calculate molecular risk at the time of diagnosis [18••]. Additional studies have used circulating tumor cells (CTCs) as an early marker of metastatic disease. The presence of CTCs has been associated with locoregional recurrence, treatment resistance, and reduced survival [19•]. However, CTC molecular characteristics that indicate patient prognosis and response to treatment are still being defined [19•, 20•].

## Innovations in Head and Neck Cancer Therapies

Apart from surgery, current HNSCC treatments include radiation, chemotherapy, and immunotherapy. Increased drug resistance to common chemotherapies has driven recent advancements. These include the repurposing of FDA-approved drugs as well as the development of novel therapies, particularly in the field of immunotherapy.

Cisplatin is the first-line chemotherapy agent used for advanced-stage HNSCC patients. This drug can be used either as a single agent for patients with low performance or in combination with radiotherapy for those with good performance [21]. However, significant resistance to this drug has resulted in reduced patient survival. Although the mechanism of cisplatin resistance is not fully understood, studies indicate that DNA methylation plays an important role. Indeed, the distinct methylation patterns associated with cisplatin-sensitive and cisplatin-resistant tumors have enabled their use as biomarkers of cisplatin-resistance [22]. This has led to the repurposing of drugs that alter the methylation of DNA in tumors. Decitabine is one such drug and has been used in clinical trials to hypomethylate the DNA of hematological and solid malignancies [23, 24]. In the context of HNSCC, preclinical data has shown that decitabine restores cisplatin sensitivity, inhibits tumor growth, and reduces cancer-related pain.

Cetuximab is another conventional chemotherapy with increased drug resistance. This chimeric monoclonal antibody binds and inhibits epidermal growth factor receptors (EGFR), which are highly expressed in the head and neck and have been repeatedly linked to HNSCC progression. Since EGFR activation stimulates cell growth, migration and survival, cetuximab’s inhibitory effects have significantly improved patient survival, especially when combined with other chemotherapy drugs [25]. Cetuximab is currently used to treat recurrent or metastatic HNSCC. However, its increasing resistance is a concern. Indeed, recent data shows that cetuximab’s efficacy, when combined with radiation therapy to treat advanced locoregional HNSCC, is inferior to cisplatin [26•]. Although numerous pathways have been linked to cetuximab resistance, the exact mechanisms remain unclear and this area warrants further research.

### Advances in Immunotherapy

Two immunotherapy drugs, nivolumab and pembrolizumab, are currently approved for HNSCC treatment. Their mechanism of action is to bind and inhibit programmed cell death protein 1 (PD-1), a protein naturally found on immune cells that regulates self-tolerance and prevents autoimmune disease by suppressing T-cell activity. In the context of cancer, PD-1 plays an important role in enabling tumor cells to evade immune attack. By inhibiting this receptor, nivolumab and pembrolizumab release T-cell suppression and promote host immune attack against cancerous cells. These two immunomodulatory agents have been shown to improve patient outcomes and increase survival rates, especially when used in conjunction with radio- or chemotherapy [27]. However, at present, they are only used in the context of recurrent or metastatic cancer and their clinical efficacy has been less than ideal (12–20% of HNSCC cases). It has been theorized that the reduced efficacy of these drugs is due, at least in part, to the immunosuppressive tumor immune microenvironment (TIME) that has been well-characterized in HNSCC [28••]. To combat this challenge, biomaterial-based drug delivery mechanisms have been developed to locally regulate the TIME and provide spatiotemporal control over the release of immunotherapeutic agents [10].

### Biomaterial Innovations Improve the Efficacy of Immunotherapies

Numerous studies have demonstrated the improved efficacy of immunotherapies when delivered via biomaterials. A prime example of this is the use of STINGel to deliver synthetic cyclic dinucleotides (CDNs), a new class of immunotherapy drugs that has been shown to strongly induce an anti-tumor immune response by activating the Stimulator of Interferon Genes (STING) pathway [29]. CDNs have shown significant therapeutic promise and have even been labelled as “intratumoral in situ vaccines” due to their ability to convert “cold tumors” with an immunosuppressive TIME, into “hot tumors” with an immunosupportive TIME. Yet, despite the therapeutic potential of these drugs, their efficacy has been poor in HNSCC preclinical models where numerous CDN doses as well as combined treatment with immune checkpoint antibodies have been required [30]. Leach et al. developed STINGel as a peptide hydrogel-based platform for the controlled intratumoral release of CDNs. Through its use of MultiDomain Peptides (MDP), STINGel is a syringe-deliverable hydrogel that self-assembles to form nanofibrous matrices. Preclinical HNSCC murine models have demonstrated that STINGel’s localized delivery of CDNs improved overall survival compared to controls [31].

The improved survival seen with STINGel was further increased following an additional iteration of the matrix design, in which the biomaterial itself was developed to serve as an immunotherapeutic agent. Inducible nitric oxide synthase (iNOS) is a pro-tumorigenic enzyme that is highly upregulated in several cancers and is known to encourage tumor growth by promoting the activation of immunosuppressive tumor-infiltrating myeloid-derived suppressor cells (MDSCs) [32–34]. While STINGel showed modest improvements in survival in previously mentioned preclinical studies, it was noted that this system failed in preclinical models with significant MDSCs. Since STINGel only addresses immune stimulation and not immune suppression, it was hypothesized that its efficacy could be improved by simultaneously targeting iNOS. The small molecule drug, *N*6-(1-iminoethyl)-l-lysine (L-NIL), is known to selectively inhibit iNOS and its downstream effects on tumor growth [32]. Rather than encapsulating this drug within the biomaterial matrix, an L-NIL drug-mimicking hydrogel (LNIL-MDP) was developed. This novel design allowed the biomaterial to combat immune suppression, with a bioactivity comparable to that of L-NIL, without the addition of any external agents [35]. Moreover, upon loading the LNIL-MDP hydrogel with CDNs to create a “SynerGel,” this biomaterial was shown to improve survival, inhibit iNOS, and affect tumor biology for an extended period of time [36••].

This concept of designing biomaterials as immunotherapeutic agents has been used by other studies. Mesoporous silica rod (MSR)-based biomaterial vaccines have been repeatedly shown to not only provide a microenvironment that supports and modulates immune cell activity in vivo but to also confer long-lasting immunity and prevent tumor recurrence [37]. Dharmaraj et al. published a key study investigating the efficacy of an MSR-based cancer vaccine targeted against HPV-16 E7 [38]. Using multiple orthotopic models of OSCC, they showed that MSR-based vaccines delayed tumor growth and prolonged survival [38].

Together, this data demonstrates feasibility and the significant clinical potential of using biomaterial-based immunotherapies to reverse the immunosuppressive TIME, generate a potent anti-tumor effect in situ, and confer long-term resistance to cancer recurrence.

## Conclusions

Despite the many inherent challenges of combating HNSCC, significant advances have been made at all stages of patient care (Fig. 1). Early-stage cancer patients can now be risk-stratified using metrics that integrate molecular biomarker data with clinicopathologic features, resulting in more accurate treatment decision. Non-invasive biopsy techniques, such as brush swabs, have enabled earlier and more frequent tissue sampling for initial risk analysis as well as in monitoring recurrence and response to treatment. Drug repurposing of demethylating drugs is being used to reverse cisplatin-resistance. In addition, injectable biomaterials are reducing systemic toxicity and improving treatment response not only through localized, controlled-release of immunotherapies but also by serving as drug mimetics themselves. The studies reviewed here highlight the critical role of interdisciplinary collaboration and the profound discoveries that can result from these endeavors. Future developments are anticipated in the use of biomarkers to monitor treatment response and the development of novel biomaterials for the simultaneous controlled-release of numerous immunotherapeutic agents.
